# Modeling the Amount of Waste Generated by Households in the Greater Accra Region Using Artificial Neural Networks

**DOI:** 10.1155/2021/8622105

**Published:** 2021-08-14

**Authors:** Charlotte Chapman-Wardy, Louis Asiedu, Kwabena Doku-Amponsah, Felix O. Mettle

**Affiliations:** Department of Statistics and Actuarial Science, School of Physical and Mathematical Sciences, University of Ghana, Legon, Accra, Ghana

## Abstract

Waste can be defined as solids or liquids unwanted by members of the society and meant to be disposed. In developing countries such as Ghana, the management of waste is the responsibility of the metropolitan authorities. These authorities do not seem to have effective management of the waste situation, and therefore, it is not unusual to see waste clog the drains and litter the streets of the capital city, Accra. The impact of waste on the environment, along with its associated health-related problems, cannot be overemphasized. The Joint Monitoring Programme report in 2015 ranked Ghana as the seventh dirtiest country in the world. The lack of effective waste management planning is evident in the large amount of waste dumped in open areas and gutters that remains uncollected. In planning for solid waste management, reliable data concerning waste generation, influencing factors on waste generation, and a reliable forecast of waste quantities are required. This study used two algorithms, namely, Levenberg–Marquardt and the Bayesian regularization, to estimate the parameters of an artificial neural network model fitted to predict the average monthly waste generated and critically assess the factors that influence solid waste generation in some selected districts of the Greater Accra region. The study found Bayesian regularization algorithm to be suitable with the minimum mean square error of 104.78559 on training data and 217.12465 on test data and higher correlation coefficients (0.99801 on training data, 0.99570 on test data, and 0.99767 on the overall data) between the target variables (average monthly waste generated) and the predicted outputs. House size, districts, employment category, dominant religion, and house type with respective importance of 0.56, 0.172, 0.061, 0.027, and 0.026 were found to be the top five important input variables required for forecasting household waste. It is recommended that efforts of the government and its stakeholders to reduce the amount of waste generated by households be directed at providing bins, increasing the frequency of waste collection (especially in highly populated areas), and managing the economic activities in the top five selected districts (Ledzekuku Krowor, Tema West, Asheidu Keteke, Ashaiman, and Ayawaso West), amongst others.

## 1. Introduction

According to Gentil et al. [1], the amount of municipal solid waste generated in many countries has been increasing for many years and this has necessitated the establishment of waste-related policies by governmental agencies and international organisations to reduce the environmental impacts of poor waste management, including reducing the amounts of waste.

Solid waste management involves holistically planning and executing the processes involved in the collection, disposal, and treatment of solid waste [2].

Solid waste management in Ghana has been analyzed from different angles by different researchers. Boadi and Kuitunen [3] studied municipal solid waste management in the Accra Metropolitan Area. They highlighted the problems that existed in the various levels of waste management in the area, namely, waste collection, disposal, and recycling. They found the waste management service woefully inadequate and recommended private sector participation. They also recommended the collation of reliable data on rubbish generation.

Miezah et al. [4] confirmed that reliable data on waste generation were nonexistent and therefore proceeded to measure the regional household generation rate and solid waste composition. They concluded that waste generation in Ghana was on the average 0.47 kg per person per day. The generation rate differed in the various regions with the coastal and forest zones generating more waste than the Northern and Savannah zones. They also found that the organic fraction constituted 49–68 percent of the waste stream.

Generally, in planning for solid waste management, what is fundamentally required is reliable data concerning waste generation, influencing factors on waste generation, and a reliable forecast of waste quantities [5, 6]. Accurate data on waste generation and factors influencing it would aid in estimating the number of waste bins to be supplied, the number of periodic collections to be done, and the landfill sites needed to be made available within a particular period.

It is evident from the above literature that planning and design of an effective municipal solid waste management system require an accurate prediction of solid waste generation.

Dyson and Chang [7] presented a new approach called “System Dynamics Modeling” for the prediction of solid waste generation in a fast-growing urban area based on a set of limited samples. By yielding more precise estimates, their proposed model outperformed the traditional least-squares regression model. Their new forecasting approach covered a variety of possible causative models and tracked inevitable uncertainties down, whereas the traditional statistical least-squares regression methods were unable to handle such issues. The limitation, however, of their proposed method is that dynamic systems models may be complex due to the combinations of simpler submodels linked to simulate the system. Their complexity is not only because all factors are simultaneously involved and affected by each other but also because they dynamically occur over time. Also, they failed to explore the concept of feedback within the system due to the difficulty of linking waste generation directly back to consumption activities.

Asante-Darko et al. [8] proposed a Fourier series model to forecast solid waste generation in Kumasi, Ghana. Their approach incorporated some characteristics of the monthly waste data for forecasting solid waste. A one-year forecast from their model revealed that the generation of solid waste will increase as a result of the high rate of urbanization and population growth. Specifically, the Fourier series model was found to have relatively smaller sum of squares error (SSE) of 1.8124 × 10^7^ at period 42, making it superior to the ARIMA times series model in the prediction of solid waste generated.

There have been a series of models developed in the literature for the prediction of waste generation. A holistic review of models developed in the literature from 2005 to 2014 revealed that the most predominant models were deterministic statistical models (multiple regression analysis, time series analysis, and some descriptive/inferential statistics) [9, 10]. These models expressed only cause-and-effect and are only effective in forecasting the amount of waste generated in a short term.

There are limited studies which adopted some artificial intelligent systems/models to predict the quantity of waste generated in a locality. One of such studies is the work of Ali Abdoli et al. [11] which used artificial neural networks to predict solid waste generation in the long term. Their study found population size, household income, and maximum temperature as effective factors in solid waste generation. Their results also revealed that the ANN model (specifically the multilayer perception) outperformed the multivariate regression model, yielding a relatively lower mean squared error (MSE) of 0.26, mean absolute percentage error (MAPE) of 0.046, and a higher correlation coefficient (*R*) of 0.86.

Kannangara et al. [12] developed models for accurate prediction of municipal solid waste (MSW) generation and diversion based on demographic and socioeconomic variables, with planned application of generating Canada-wide MSW inventories. Two machine learning algorithms, namely, decision trees and neural networks, were applied to build the models. Their results showed that machine learning algorithms can successfully be used to generate waste models with good prediction performance. Specifically, the neural network models had the best performance, describing 83% of the variation in the training data and 72% of the variation in the test data with in-sample and out-of-sample prediction errors of 15% and 16%, respectively.

As indicated by Liu and Yu [13], a better understanding of the factors that affect the generation of municipal living refuse and the accurate prediction of its generation are crucial for municipal planning projects and city management. Most of the studies in the literature described some challenges of the solid waste management with recommendations, whereas the very few that modeled the quantity of waste generated mainly used deterministic techniques with some isolated machine learning models. In this study, we harness the predictive power of artificial neural networks to model the quantity of waste generated in some selected municipalities in Ghana by assessing some critical waste-generating factors.

The rest of the paper is organized as follows: Section 2 (Materials and Methods) discusses the mathematical foundations of the adopted algorithms in estimating the neural network model parameters and the evaluation criteria used to assess the algorithms.  Section 3 (Results and Discussion) presents and discusses the results of the ANN model and assesses the critical factors used in the prediction of the household solid waste generated.  Section 4 examines the findings of the study in comparison with existing works in the literature and finally concludes by summarizing the overall achievements of the study. The section also presents some recommendations and directions for future developments.

## 2. Materials and Methods

### 2.1. Description of Data

Primary data were obtained from households. These data contained information on the independent variables that had influence on solid waste generated in the districts.

To ascertain the critical factors that influence solid waste generation in the Greater Accra region, information was solicited from various households in the region. Information from the households was used to investigate the effect of sociodemographic variables such as age, house type, educational level, religion, residency type, household size, employment category, household waste disposal method, frequency of waste collection, and income levels on solid waste generation in the region.

The study sample of the households was drawn from fifteen of the twenty-six districts in the Greater Accra region. A two-stage sampling approach was used in this study. The approaches comprised stratification with proportional allocation to size and simple random sampling. The randomly selected districts were considered homogeneous units. This was because, with reference to the stratification variable (the amount of solid waste generated), it was expected that the districts would be homogeneous units due to the type of economic activities that exist in the district. Each district therefore represented a stratum.

A sample size of 2102 households was used for the study. This is a representative sample which can be used to make inference about the population on households in the Greater Accra Metropolis with 2.5% margin of error. The average monthly waste for each sampling unit (household) was also recorded to be used as the target variable.

Seventy percent (70%) of the data was used for training the algorithm, while the remaining 30% was used for testing and validation.  Table 1 shows the various input variables and the target variables used in this study.

### 2.2. Artificial Neural Networks (ANNs)

According to Gershenson [14], ANNs are named after the neurons in the human brain. They are a set of algorithms modeled like the human nervous system and are designed to recognize patterns and relationships in data. They work as neurons in the human body, in that they receive stimuli, work on them, and transmit them to other processing units. Dike et al. [15] identified three learning methods in ANNs; they are unsupervised, supervised, and reinforced learning methods. Unsupervised learning occurs when there is a predictor variable *X* with no corresponding labeled output variable. Unsupervised learning is able to solve association and clustering problems [16]. Supervised learning involves an input variable *X* and an output variable *Y*. This method of learning is suitable for solving classification and regression problems [16].

Reinforcement learning as described by Dike et al. [15] learns through interconnections with the environment and is usually demonstrated as a Markov decision process. ANNs are mainly categorized by their architecture. The three main types of neural network architecture are feed forward, recurrent [17], and convolutional neural networks [18].

The architecture is called feed forward because the flow of information takes place in the forward direction. A feed forward network defines a mapping *y*=*f*(*x*; **p**) and learns the value of the parameters **p** that result in the best function approximation [19].

A feed forward can either be single layered or multilayered. The single-layer network consists of only one hidden layer.

The multilayered neural network is also known as the deep learning network. The distinguishing feature of this network is the fact that it has multiple hidden layers for complex processing. The hidden layer can be seen as a *distillation layer* that distills some of the important patterns from the inputs and passes them onto the next layer [20]. The ANN architecture best suited to the data and therefore adopted for the study is the feed forward multilayered neural network. The network structure as earlier described includes multiple hidden layers for complex processing which involves estimating the parameters **p** that result in the best function approximation.

In this study, we adopted two algorithms (Levenberg*–*Marquardt (LM) and Bayesian regularization (BR)) for solving the nonlinear equations that resulted in the estimation of the parameters **p**.

#### 2.2.1. Levenberg–Marquardt (LM) Algorithm

In fitting a model $\hat{y}\left(X,p\right)$ of an independent variable *X* and a vector of *n* parameters **p** to a set of *m* data points (*x*_*i*_, *y*_*i*_),  *i*=1,2,…, *m*, the convention is to minimize the sum of weighted squares of the errors between the data *y*_*i*_ and the fitted curve with function $\hat{y}\left(X,p\right)$.

The expression which represents the weighted residuals (weighted squares of errors) between *y*_*i*_ and $\hat{y}\left(X,p\right)$ is the error function given as

(1) $$\begin{array}{c} \chi_{m}^{2}\left(p\right)=\sum_{i=1}^{m}{\left[\frac{y\left(x_{i}\right)-\hat{y}\left(x_{i},p\right)}{\sigma_{y_{i}}}\right]}^{2}\\ ={\left(y-\hat{y}\left(p\right)\right)}^{T}W\left(y-\hat{y}\left(p\right)\right)\\ =y^{T}Wy-2y^{T}W\hat{y}+{\hat{y}}^{T}W\hat{y}. \end{array}$$

As $\left(y\left(x_{i}\right)-\hat{y}\left(x_{i},p\right)/\sigma_{y_{i}}\right)∼N\left(0,1\right)$, it follows that ${\left[y\left(x_{i}\right)-\hat{y}\left(x_{i},p\right)/\sigma_{y_{i}}\right]}^{2}∼\chi_{1}^{2}\left(p\right)$ and therefore $\sum_{i=1}^{m}{\left[y\left(x_{i}\right)-\hat{y}\left(x_{i},p\right)/\sigma_{y_{i}}\right]}^{2}∼\chi_{m}^{2}\left(p\right)$.

If *σ*_*y*_*i*__ represents the standard error for the *i*th data point (*x*_*i*_, *y*_*i*_), then **W** = (*w*_*ii*_), *i*=1,2,…, *m*, is a diagonal weight matrix with the *i*th diagonal entry *w*_*ii*_ = 1/*σ*_*y*_*ii*__^2^.

Suppose the function $\hat{y}\left(X,p\right)$ is nonlinear with the model parameters **p**; then, a reduction of the error function *χ*^2^(**p**) can only be obtained iteratively. The goal of each iteration will be to find a small change *δ* in the parameters **p** that reduces *χ*^2^(**p**).

LM is one of many algorithms used to perform the iteration. LM is said to be made up of two processes, namely, the gradient descent and Gauss–Newton methods.

The LM algorithm can be represented as follows:

(2) $$\begin{array}{c} \left[J^{T}WJ+\mu I\right]\delta_{lm}=J^{T}W\left(y-\hat{y}\right), \end{array}$$

where *δ*_*lm*_ is the change in parameters due to the LM algorithm. Also,

 
*μ* is a strictly positive scalar referred to as the damping term [21]
 
**I** is the identity matrix and $J=\partial \hat{y}\left(p\right)/\partial p$ (the matrix whose entries are the partial derivatives of $\hat{y}\left(p\right)$ with respect to the parameters **p**)

In LM, the damping term is adjusted at each iteration. When **p** is far from the solution, the damping term is set to a large value and as a result, the LM algorithm approaches the gradient descent algorithm. This is because

(3) $$\begin{array}{c} \delta_{lm}={\left[J^{T}WJ+\mu I\right]}^{-1}\times J^{T}W\left(y-\hat{y}\right), \end{array}$$

where [**J**^*T*^**WJ**+*μ ***I**]^−1^ represents the length of the step in gradient descent.

As the values of **p** approach the required solution, the damping term is reduced and the LM algorithm approaches the Gauss–Newton algorithm. This is because [**J**^*T*^**WJ**+*μ ***I**] ≈ [**J**^*T*^**WJ**].

*(1) Converging Criteria*. According to Lourakis et al. [22], iteration will progress until one of the following criteria is met:

- There is convergence in gradient. That is, $\left|J^{T}W\left(y-\hat{y}\right)\right|<\epsilon_{2}$.
- The relative change in the magnitude of *μ* drops below a threshold *ε*_3_.
- The error *ξ*^*T*^*ξ* falls below a threshold *ϵ*_4_.
- The maximum number of iterations is completed.

#### 2.2.2. Bayesian Regularization (BR) Algorithm

In applying the Bayesian concept to the regression framework, assume an underlying functional model

(4) $$\begin{array}{c} y_{i}=f\left(x_{i}:W\right)+\varepsilon_{i}. \end{array}$$

If we assume that the *ϵ*_*i*_ ~ *N*(0, *σ*^2^), then it follows that

(5) $$\begin{array}{c} P\left(y_{i}|x_{i},W,\sigma^{2}\right)=N\left(f\left(x_{i}:W\right),\sigma^{2}\right). \end{array}$$

Given that the **x**_*i*_′*s* is mutually independent, the likelihood of all the data observed will therefore be

(6) $$\begin{array}{c} =\prod_{i=1}^{n}P\left(y_{i}|x_{i},W,\sigma^{2}\right)\\ =\prod_{i=1}^{n}{\left(\frac{1}{2\pi \sigma^{2}}\right)}^{-\left(1/2\right)}\exp\left[\frac{{\left(y_{i}-W,x_{i}\right)}^{2}}{2\sigma^{2}}\right]. \end{array}$$

The Bayesian prior specifies the belief about the parameter to be determined. According to Thanh et al. [23], we may choose a zero-mean Gaussian prior and introduce another parameter which controls the strength of our belief about the parameter **W**. The prior can therefore be given as

(7) $$\begin{array}{c} P\left(W|\alpha \right)=\prod_{i=1}^{m}{\left(\frac{\alpha }{2\pi }\right)}^{1/2}\exp\left[\frac{\alpha }{2}W^{2}\right]. \end{array}$$

Now, we consider the normalizing constant or the evidence used in updating one's belief about the parameter. This is normally a constant which is usually ignored in the posterior calculations.

By applying the Bayesian concept, the posterior distribution will be expressed as

(8) $$\begin{array}{c} P\left(W|x_{i},y,\alpha ,\sigma^{2}\right)=\frac{P\left(y|W,x_{i},\sigma^{2}\right)P\left(W|\alpha \right)}{P\left(y|\alpha ,\sigma^{2}\right)}. \end{array}$$

As *P*(**y***|α*, *σ*^2^) is a constant, equation (8) can be represented as

(9) $$\begin{array}{c} P\left(W|x_{i},y\alpha ,\sigma^{2}\right)\propto P\left(y|W,x_{i},\sigma^{2}\right)P\left(W|\alpha \right)\\ \propto \prod_{i=1}^{n}{\left(\frac{1}{2\pi \sigma^{2}}\right)}^{1/2}\exp\left[\frac{-{\left(y_{i}-w_{i}x_{i}\right)}^{2}}{2\sigma^{2}}\right]\times \prod_{i=1}^{n}{\left(\frac{\alpha }{2\pi }\right)}^{1/2}\exp\left[-\frac{\alpha }{2}w_{i}^{2}\right]\\ \propto {\left(\frac{1}{2\pi \sigma^{2}}\right)}^{n/2}\exp\left[-\frac{1}{2\sigma^{2}}\sum_{i=1}^{n}{\left(y_{i}-w_{i}x_{i}\right)}^{2}\right]\times {\left(\frac{\alpha }{2\pi }\right)}^{n/2}\exp\left[-\frac{\alpha }{2}\sum_{i=1}^{n}w_{i}^{2}\right]\\ =K\;\exp\left[-\frac{1}{2\sigma^{2}}\sum_{i=1}^{n}{\left(y_{i}-w_{i}x_{i}\right)}^{2}-\frac{\alpha }{2}\sum_{i=1}^{n}w_{i}^{2}\right]\\ =K\;\exp\left[-\frac{1}{2\sigma^{2}}{\left(y-Wx\right)}^{T}\left(y-Wx\right)-\frac{\alpha }{2}W^{T}W\right]\\ =K\;\exp\left[-\frac{1}{2\sigma^{2}}\left(y^{T}y-y^{T}Wx-x^{T}W^{T}y+x^{T}W^{T}Wx\right)-\frac{\alpha }{2}W^{T}W\right]\\ =A\;\exp\left[-\frac{1}{2\sigma^{2}}\left(-2W^{T}x^{T}y+W^{T}x^{T}xW\right)-\frac{\alpha }{2}W^{T}W\right]\\ =A\;\exp\left\{-\frac{1}{2\sigma^{2}}\left[-2W^{T}x^{T}y+W^{T}x^{T}xW+\sigma^{2}\alpha W^{T}W\right]\right\}\\ =A\;\exp\left\{-\frac{1}{2\sigma^{2}}\left[W^{T}\left(x^{T}x+I\sigma^{2}\alpha \right)W-2W^{T}x^{T}y\right]\right\}, \end{array}$$

where *K*=(*α*/4*π*^2^*σ*^2^)^*n*/2^ and *A*=*K*  exp{−(1/2*σ*^2^)(**y**^*T*^**y**)}.

Completing the squares of the right hand side of equation (9), we obtain

(10) $$\begin{array}{c} A\;\exp\left\{-\frac{1}{2\sigma^{2}}\left(x^{T}x+I\sigma^{2}\alpha \right){\left[W-{\left(x^{T}x+I\sigma^{2}\alpha \right)}^{-1}x^{T}y\right]}^{T}\left[W-{\left(x^{T}x+I\sigma^{2}\alpha \right)}^{-1}x^{T}y\right]\right\}. \end{array}$$

We can therefore conclude that **W** ~ *N*_*m*_(*μ*, Σ) (a multivariate normal distribution) is specified as

(11) $$\begin{array}{c} \mu ={\left(x^{T}x+I\sigma^{2}\alpha \right)}^{-1}x^{T}y,\\ \Sigma =\sigma^{2}{\left(x^{T}x+I\sigma^{2}\alpha \right)}^{-1}. \end{array}$$

Now,

(12) $$\begin{array}{c} P\left(y|\alpha ,\sigma^{2}\right)=\int P\left(y|W,x_{i},\sigma^{2}\right)P\left(W|\alpha \right)\text{d}W\\ ={\left(2\pi \right)}^{-\left(n/2\right)}{\left[\sigma^{2}I+\alpha^{-1}x^{T}x\right]}^{-\left(1/2\right)}\exp\left[-\frac{1}{2}y^{T}{\left(\sigma^{2}I+\alpha^{-1}x^{T}x\right)}^{-1}y\right],\\ \log\;P\left(y|\alpha ,\sigma^{2}\right)=-\frac{n}{2}\log\left(2\pi \right)-\frac{1}{2}\log\left[\sigma^{2}I+\alpha^{-1}x^{T}x\right]-\left[\frac{1}{2}y^{T}{\left(\sigma^{2}I+\alpha^{-1}x^{T}x\right)}^{-1}y\right],\\ \frac{\partial \;\log P\left(y|\alpha ,\sigma^{2}\right)}{\partial \sigma^{2}}=-\frac{1}{2}I{\left(\sigma^{2}I+\alpha^{-1}x^{T}x\right)}^{-1}+\frac{1}{2}y^{T}{\left(\sigma^{2}I+\alpha^{-1}x^{T}x\right)}^{-2}y,\\ \frac{\partial \;\log P\left(y|\alpha ,\sigma^{2}\right)}{\partial \alpha }=\frac{1}{2}\left(\alpha^{-2}x^{T}x\right)\left(\sigma^{2}I+\alpha^{-1}x^{T}x\right)-\frac{1}{2}y^{T}{\left(\sigma^{2}I+\alpha^{-1}x^{T}x\right)}^{-2}y\left(\alpha^{-2}x^{T}x\right). \end{array}$$

We now solve the normal equations as follows:

(13) $$\begin{array}{c} \frac{\partial \;\log P\left(y|\alpha ,\sigma^{2}\right)}{\partial \alpha }=0,\\ \frac{\partial \;\log P\left(y|\alpha ,\sigma^{2}\right)}{\partial \sigma^{2}}=0, \end{array}$$

to obtain

(14) $$\begin{array}{c} \alpha =\frac{x^{T}x}{y^{T}y-\sigma^{2}I}. \end{array}$$

Tipping [24] introduced a parameter *γ* which determines the influence of the posterior and likelihood on *w*_*i*_. As *γ* ⟶ 0, the influence of the prior is captured and as *γ* ⟶ 1, the influence of the likelihood is captured.

*γ* is given as

(15) $$\begin{array}{c} \gamma =1-\alpha \sigma^{2}I. \end{array}$$

*(1) Inference Procedure*. Tipping [24] described the inference procedure as follows:

1. Fix *σ*^2^ and estimate *α*_*i*_ from equation (14)
2. Compute the weight posterior statistics *μ* and ∑ from equation (11)
3. Compute *γ* and reestimate *α*_*i*_ and *σ*_*i*_^2^
4. Repeat (2) and the whole process until convergence

### 2.3. Evaluation of the Study Algorithms

To evaluate the performance of the ANN models, three indices were assessed: mean square error (MSE), correlation coefficient (*R*), and coefficient of determination (*R*^2^). Other metrics such as the number numerical iteration (*n*) and the runtime of the algorithms were computed. The accuracy and suitability of the models were determined using these criteria.

The MSE is an important measure of the algorithms' precision since the adopted algorithms (BR and LM) for solving the nonlinear problems only aid in the estimation of the ANN model parameters for prediction.

The correlation coefficient measures the degree of linear association between the target variable (average waste generated) and the predicted output, whereas the coefficient of determination (*R*^2^) describes the percentage of variation in the study data explained by the fitted model. An algorithm with the minimum MSE and relatively higher *R* and *R*^2^ is preferred.

## 3. Results and Discussion

**Figure 1:**
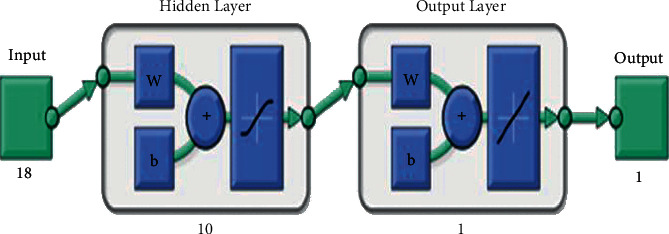
Neural network diagram.

Figure 1 presents the neural network diagram/architecture used for the study. There were 18 input variables (predominant age category, house type, educational level, religion, residential status, household size, employment category, household waste disposal method, frequency of waste collection, income levels, etc.), 10 allowed hidden neurons, and 1 target variable (average monthly waste in tonnes per household).

**Figure 2:**
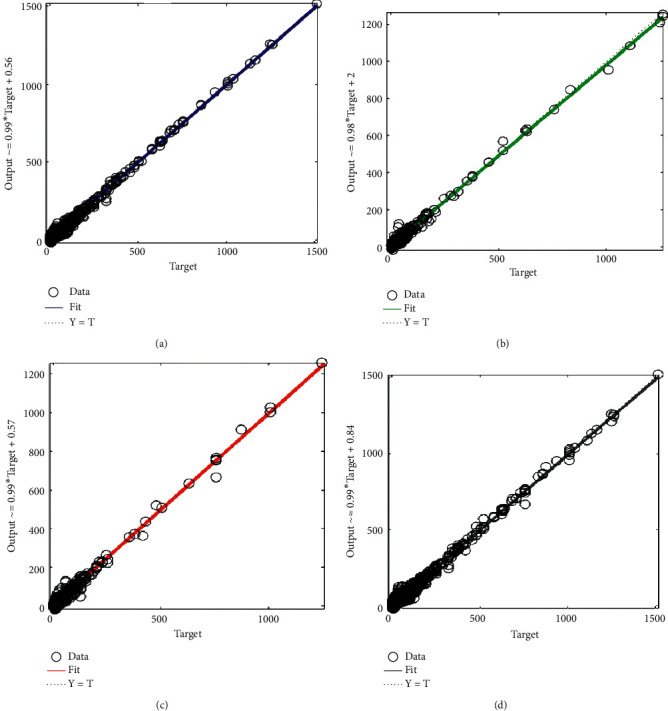
Regression plot between the target variable and the predicted output using the Levenberg–Marquardt algorithm. (a) Training: *R* = 0.99648. (b) Validation: *R* = 0.99717. (c) Test: *R* = 0.99412. (d) Overall: *R* = 0.99627.

Figure 2 shows a regression fit between the target variable and the predicted output using the Levenberg–Marquardt algorithm. The subgraphs in Figures 2(a)–2(d) show the regression fits between the target variable and the predicted output on training data, validation data, test data, and the overall data, respectively.

From Figure 2, the correlations between the target variable (average monthly household waste generated) and the predicted output are 0.99648 (for training data), 0.99717 (for validation), 0.99412 (for test data), and 0.99627 (for the overall data). This indicates a very good fit since there exists a very strong positive linear relationship (in all cases) between the target variable (monthly household waste generated) and the predicted output using the LM algorithm.

**Figure 3:**
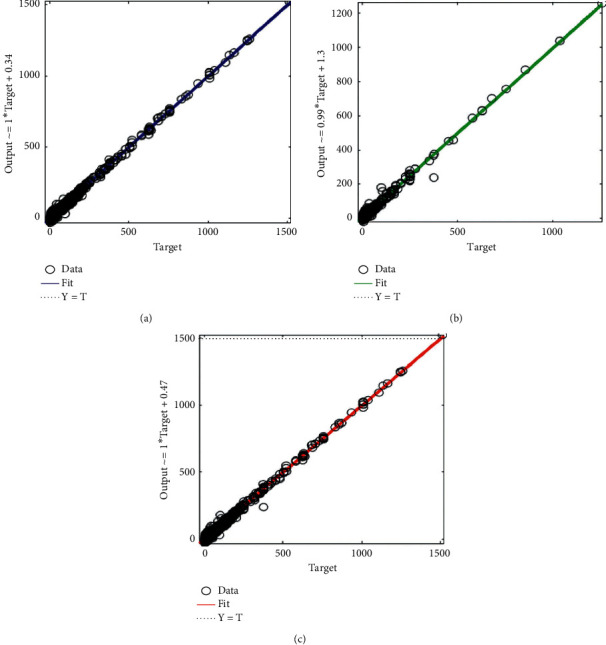
Regression plot between the target variable and the predicted output using the Bayesian regularization algorithm. (a) Training: *R* = 0.99801. (b) Test: *R* = 0.99570. (c) Overall: *R* = 0.99767.

Figure 3 shows a regression fit between the target variable and the predicted output for the training data, test data, and the overall data using the BR algorithm.

It can be seen from Figure 3 that the correlations between the target variable (average monthly household waste generated) and the predicted output are 0.99801 (for training data), 0.99570 (for test data), and 0.99767 (for the overall data). This signifies a very good fit since there exists a very strong positive linear relationship (in all cases) between the target variable (monthly household waste generated) and the predicted output using the Bayesian regularization algorithm for prediction.

**Figure 4:**
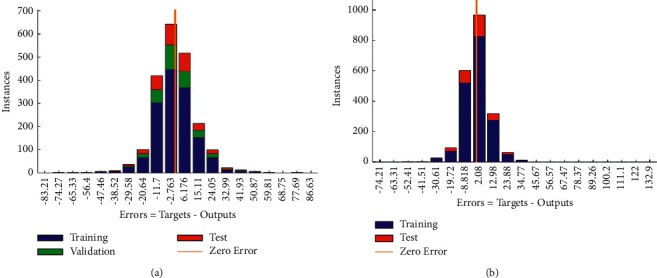
Error distribution of the study algorithms. (a) LM algorithm. (b) BR algorithm.

The error distributions of the LM and BR algorithms are shown in Figure 4.

It can be inferred from the error histogram in Figure 4 that the error distribution for both algorithms is approximately normal. This makes it suitable to generalize the predictions of the algorithms. More so, this satisfies the underlying assumption of the Bayesian regularization algorithm; that is, the errors (*ϵ*_*i*_) are expected to be normally distributed with mean 0 and a constant variance of *σ*^2^.

Table 2 contains the mean square errors (MSEs), the correlation coefficient between the target variable and the predicted output (*R*), the coefficient of determination (*R*^2^), the number of numerical iterations (*n*), and runtime of the study algorithms.

It is evident from Table 2 that the Levenberg–Marquardt (LM) algorithm recorded an MSE of 172.06769 (on training data), 211.47506 (on validation), and 285.44368 (on test data). This was slightly higher than the MSE of the Bayesian regularization algorithm which was 104.78559 (on training data) and 217.12465 (on test data).

It can be inferred from this finding that the BR algorithm has a relatively better precision in estimating the parameters of the neural network model.

Generally, the correlations between the target variable and the predicted output were relatively higher (with a correlation of 0.99801 for training/in-sample prediction and 0.99570 for testing/out-of-sample prediction) when the BR algorithm was used for estimating the neural network model parameters. It is worthy to note that the validation of the BR algorithm is inherent. This accounts for the missing values shown in Table 2.

The ANN model explained 99.30% of the variation in training data, 99.43% of the variation in the validation data, and 98.83% of the variation in test data when the LM algorithm was used to estimate the model parameters, whereas the model described 99.60% of the variation in the training data and 99.14% of the variation in test data when the BR algorithm was used to estimate the model parameters.

From the results of the coefficient of determination (*R*^2^), it can be concluded that the fitted ANN model explains relatively higher variations in the study data when the BR algorithm is used to estimate the model parameters.

The LM algorithm converged after 60 iterations with a runtime of about 1.5 seconds, whereas the BR algorithm converged after 540 iterations with a runtime of about 4 seconds. This means that the LM algorithm converges faster than the BR algorithm. This was expected as the Bayesian regularization algorithm is a more data-driven mechanism which requires more time but usually results in better generalization.

**Figure 5:**
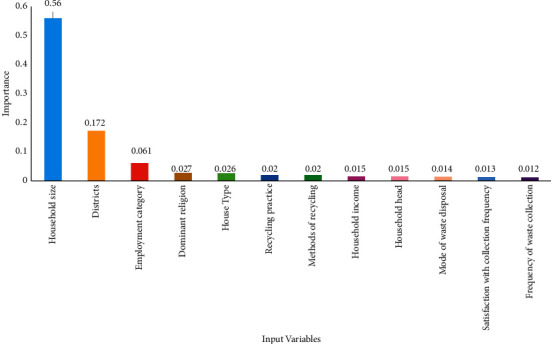
A graph of some important independent input variables.

Figure 5 shows some independent input variables ranked according to their level of importance.

It can be seen from Figure 5 that household size was the most important variable in predicting the amount of waste generated by a household. Household size recorded a variable importance value of 0.56 of 1. It turned out that highly populated households generated more waste than moderately and less populated households. The district in which a household is situated was the next important variable with a variable importance of 0.172 of 1, in predicting the amount of waste generated. The employment category (formal, informal, retired, and other) was the third most important variable in the prediction of the amount of household waste generated (0.061 of 1).

Table 3 shows the average monthly waste generated by households based on the various subgroups of the important independent variables. As stated earlier, there were 15 districts in the study sample. It can be seen from Table 3 that households in the Ledzekuku Krowor district generated the highest average monthly waste (339.3541 tonnes) with a standard error of 19.5854. This was followed by households in the Tema West district which generated a monthly average waste of 268.2114 tonnes with a standard error of 25.9443. The predominant activity in these districts is fishing, and most of the households in these districts have relatively higher household sizes. Households in the Okaikoi North district generated the lowest waste producing an average monthly waste of 5.4072 tonnes with a standard error of 0.1378. This can be attributed to the fact that households in these districts have relatively lower household sizes.

Considering the employment category variable, households whose members are predominantly formal workers generated the highest average monthly waste (87.5443 tonnes) with a standard error of 5.6529. This was closely followed by households whose members are predominantly informal workers with an average monthly waste of 81.9569 tonnes (standard error of 4.6827). This could be because households with predominantly formal workers are more likely to use the regular methods of waste disposal such as collection by waste-disposal agents, whereas the households with predominantly informal workers are more likely to use the traditional methods of disposal such as burning and burying. Waste disposed using the traditional methods could not be accounted for.

Households whose members are predominantly Christian generated the highest average monthly waste of 85.3959 tonnes with a standard error of 4.0806, followed by Muslims with an average of monthly waste of 78.7520 tonnes (standard error of 6.6658). Households whose members are from traditional and other religions were in the minority and generated the lowest waste. It is worth noting that most of the survey districts contain predominantly Christian communities.

Considering the house type variable, flat/apartments generated the highest average monthly waste of 150.0595 tonnes with a standard error of 2.4592. This was followed by semidetached households which generated an average monthly waste of 111.2652 with a standard error of 6.2138. Residents of uncompleted buildings were the third group in this category with an average monthly waste of 82.8600 tonnes (standard error of 16.8051). Standalone households tend to generate the lowest waste with an average monthly waste of 40.1873 tonnes (standard error of 9.7802). This could be attributed to the household sizes of these house types.

## 4. Conclusion and Recommendation

The study successfully assessed two algorithms (Levenberg–Marquardt and Bayesian regularization) for estimating the neural network model parameter to aid in the prediction of average monthly waste generated by households in some selected districts in the Greater Accra region of Ghana using some sociodemographic characteristics of the households. The Bayesian regularization algorithm outperformed the Levenberg–Marquardt algorithm, producing a comparatively lower MSE of 104.78559 on training data and 217.12465 on test data. The BR algorithm also gave the highest correlation coefficients (0.99801 on training data, 0.99570 on test data, and 0.99767 on the overall data) between the target variable (average monthly waste) and the predicted output. This signifies a good fit and makes Bayesian regularization a suitable and preferred algorithm estimating the ANN model parameters to aid in the prediction of average waste generated by households in the long term. Also, the fitted ANN model explains relatively higher variations in the study data when the BR algorithm is used to estimate the model parameters.

Although the LM algorithm was faster in convergence (with 60 iterations and a runtime of about 1.5 seconds) than the BR algorithm (with 540 iterations and a runtime of about 4 seconds), the BR algorithm is a more data-driven mechanism which enables generalization of the predicted outputs.

The study also revealed that household size, districts, employment category, dominant religion, and house type with a respective importance of 0.56, 0.172, 0.061, 0.027, and 0.026 were the five most important independent input variables required to predict the amount of waste generated by a household. Specifically, highly populated households generated more waste than moderately and less populated households. This result is consistent with the findings of Ali Abdoli et al. [11].

Ledzekuku Krowor, Tema West, Asheidu Keteke, Ashaiman, and Ayawaso West were the five districts (arranged in order) that generated the highest waste. This could possibly be due to the type of economic activities in the district and the population size of the districts.

The study also found households which are flat/apartment and semidetached and uncompleted buildings (arranged in order) as the top three house types which produce relatively higher waste. This could be accounted by the number of people or the household size of these house types. It is recommended that efforts of the government and its stakeholders to reduce the amount of waste generated by households be directed at providing bins, increasing the frequency of waste collection, and managing the economic activities in the top five selected districts (Ledzekuku Krowor, Tema West, Asheidu Keteke, Ashaiman, and Ayawaso West), amongst others.

Despite the prediction of the average waste generated and assessing the effect of some important critical factors, the study failed to isolate the components of the household solid waste generated. Future studies would focus on assessing the components of the household solid waste to aid in the development of effective solid waste management systems.

The ANN (with Bayesian regularization option) is also recommended as a suitable algorithm for predicting the amount of waste generated by households using some critical waste generation factors. The model can also be used in application areas that require the prediction of specified targets in the long term.

## Figures and Tables

**Table 1:** Table showing districts with number of households, proportional rates, and sample size.

District	House type	Res. status	Comm. type	Household size	Religion	⋯	Waste (tonnes)
1	1	1	1	4	1		7.6472
1	1	2	2	6	1		11.4708
1	3	1	3	13	1		24.8534
⋮	⋮	⋮	⋮	⋮	⋮	⋯	⋮
2	2	3	2	6	1		60.7554
2	1	3	2	5	1		50.6295
2	4	1	2	20	1		202.518
⋮	⋮	⋮	⋮	⋮	⋮	⋯	⋮
3	2	2	2	10	1		106.885
3	5	3	3	8	2		85.508
3	4	2	3	35	2		374.0975
⋮	⋮	⋮	⋮	⋮	⋮	⋯	⋮
4	4	2	3	20	1		319.672
4	4	2	3	4	1		63.9344
4	3	1	3	6	1		95.9016
⋮	⋮	⋮	⋮	⋮	⋮	⋯	⋮
9	3	1	2	5	1		24.727
9	4	2	2	6	1		29.6724
9	3	1	2	5	1		24.727
⋮	⋮	⋮	⋮	⋮	⋮	⋯	⋮
15	2	2	1	5	1		103.9705
15	2	1	1	9	1		187.1469
15	4	2	2	20	1	⋯	415.882

Districts: (1) Ablekuma, (2) Adenta, (3) Ashiedu Keteke, (9) Kpone Katamaso, (10) La Dade-Kotopon, and (15) Tema Metropolitan. House type: (1) standalone, (2) semidetached, (3) flat/apartment, (4) compound house/single room, (5) tent/kiosk/containers, and (6) uncompleted buildings. Residency type: (1) self-ownership, (2) rental, and (3) other. Community type: (1) high income, (2) medium income, and (3) low income. Religion: (1) Christians, (2) Muslims, (3) traditional, (4) atheists, and (5) other. Source: primary data collection.

**Table 2:** Evaluation of the study algorithms.

		Samples	MSE	*R*	*R*^2^(%)	*n*	Runtime (sec.)
LM	Training	1472	172.06769	0.99648	99.30	60	1.5
	Validation	315	211.47506	0.99717	99.43		
	Testing	315	285.44368	0.99412	98.83		

BR	Training	1472	104.78559	0.99801	99.60	540	4
	Validation	315	—	—	—		
	Testing	315	217.12465	0.99570	99.14		

**Table 3:** Descriptive statistics of some important independent variables.

Input variable	Categories	*N*	Average waste	Std. error
Districts	Ledzekuku Krowor	218	339.3541	19.5854
	Tema West	128	268.2114	25.9443
	Asheidu Keteke	145	98.1131	5.7312
	Ashaiman	127	95.1465	7.3129
	Ayawaso West	108	71.6173	6.1112
	Kpone Katamanso	140	50.5609	1.7668
	Adenta	56	43.0351	3.8554
	La Nkwantanang	152	35.8380	1.0795
	Tema East	100	35.1688	2.1869
	Ablekuma	162	26.8360	1.3132
	La Dade-Kotopon	136	25.3088	0.8195
	Korle Klottey	158	17.1120	0.9357
	Ayawaso East	159	16.6744	0.4495
	Okaikoi South	150	10.5041	0.5572
	Okaikoi North	163	5.4072	0.1378

Employment category	Formal	959	87.5443	5.6529
	Informal	1071	81.9569	4.6827
	Retired (formal)	54	78.4510	12.6877
	Retired (informal)	11	28.8794	7.7272
	Others	7	17.7322	6.4310

Dominant religion	Christian	1716	85.3959	4.0806
	Muslim	367	78.7520	6.6658
	Traditional	15	62.7918	9.6645
	Other	4	6.4682	0.7909

House type	Flat/apartment	497	150.0595	2.4592
	Semidetached	296	111.2652	6.2138
	Uncompleted building	315	82.8600	16.8051
	Compound/single room	822	81.4325	5.3214
	Tent/kiosk/containers	137	46.6797	3.8222
	Standalone	35	40.1873	9.7802

## Data Availability

The .xlsx data used to support the findings of this study are available from the corresponding author upon request.
